# Supplementary material for: De novo design of protein minibinder agonists of TLR3
Source: bioRxiv. 2024 Apr 18:2024.04.17.589973. Preprint. [Version 1] doi: 10.1101/2024.04.17.589973 (PMC11042314; doi:10.1101/2024.04.17.589973)

# Minibinder 1

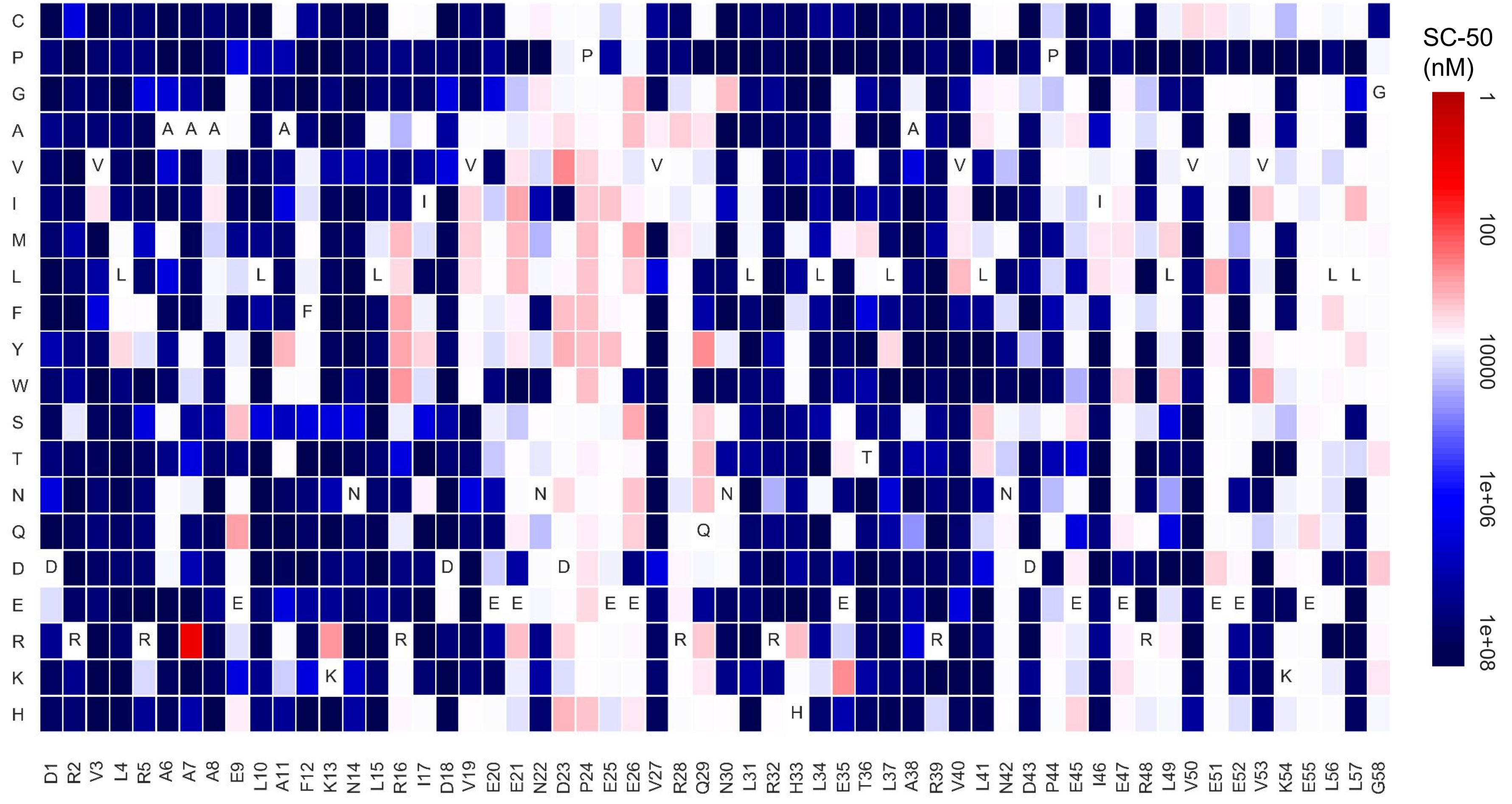

# Minibinder 2

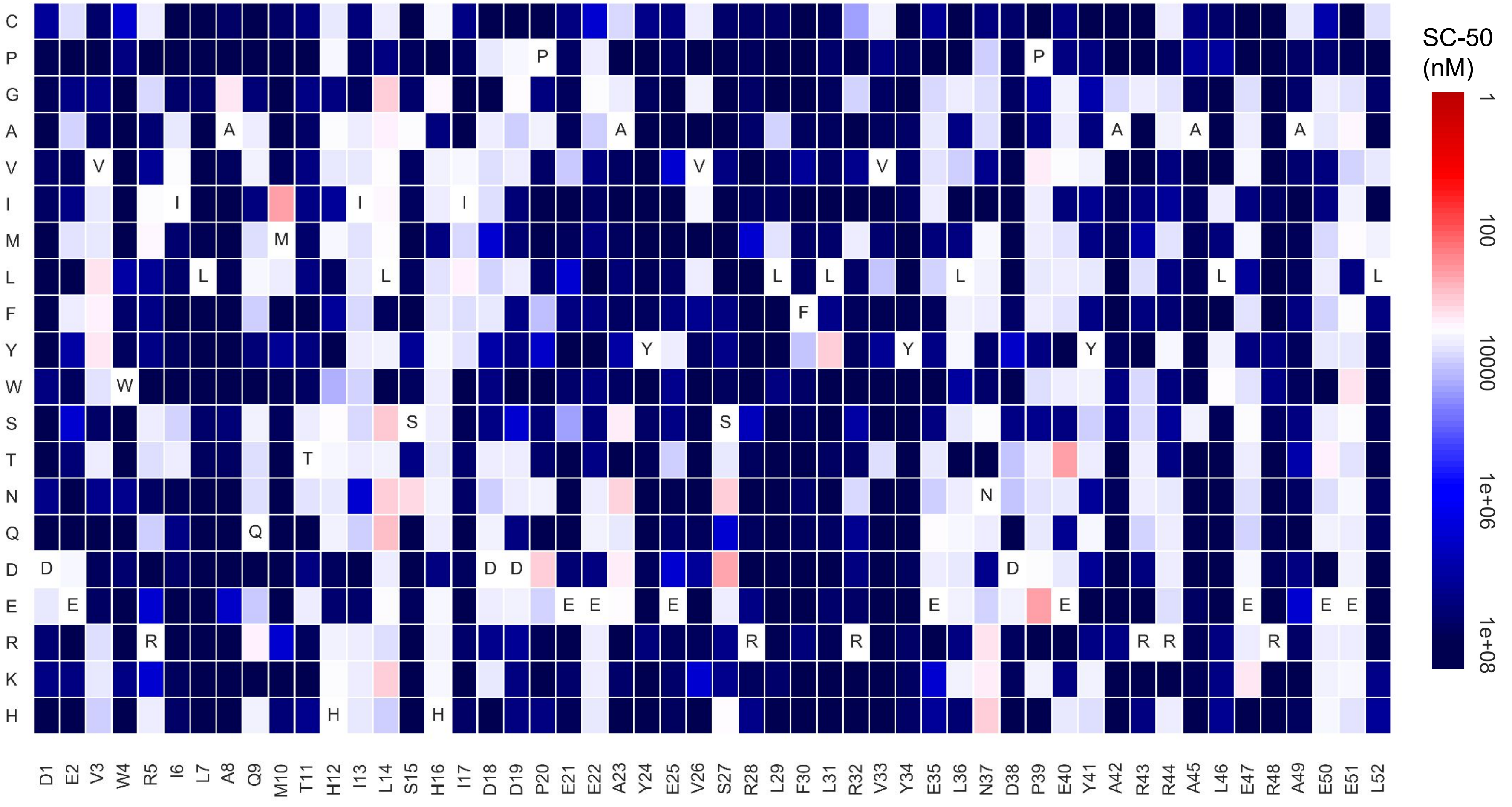

# Minibinder 3

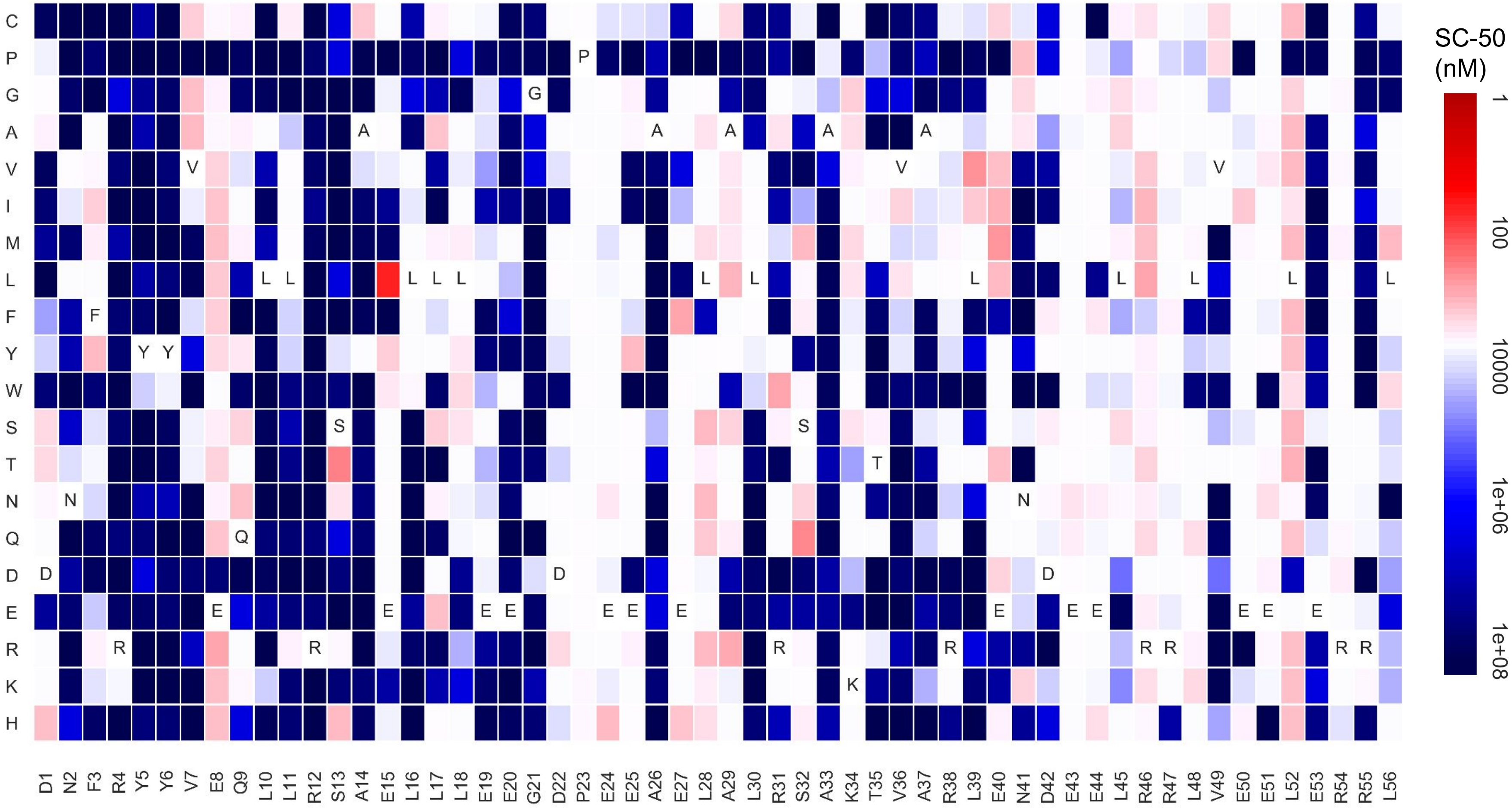

# Minibinder 4

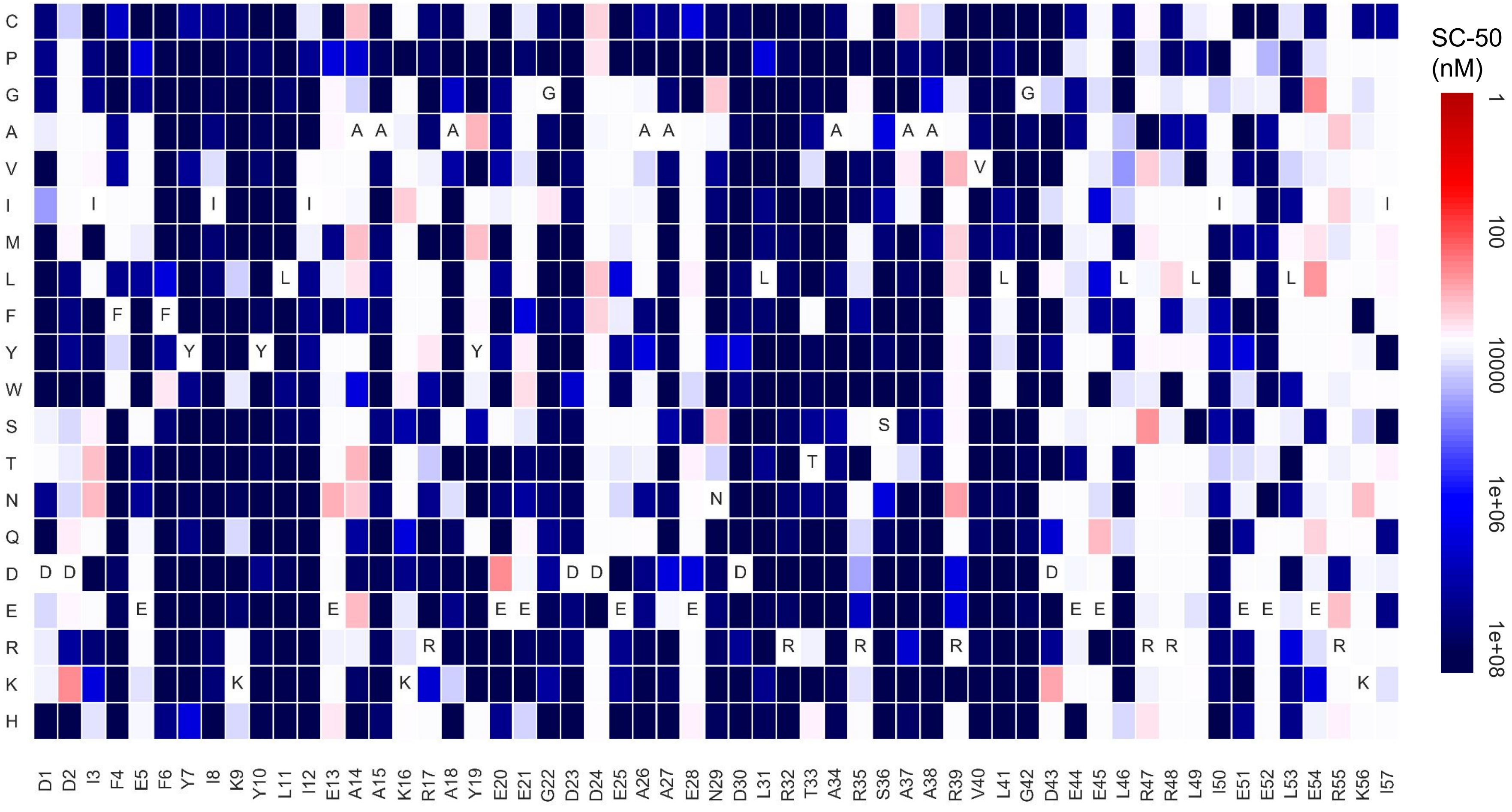

# Minibinder 5

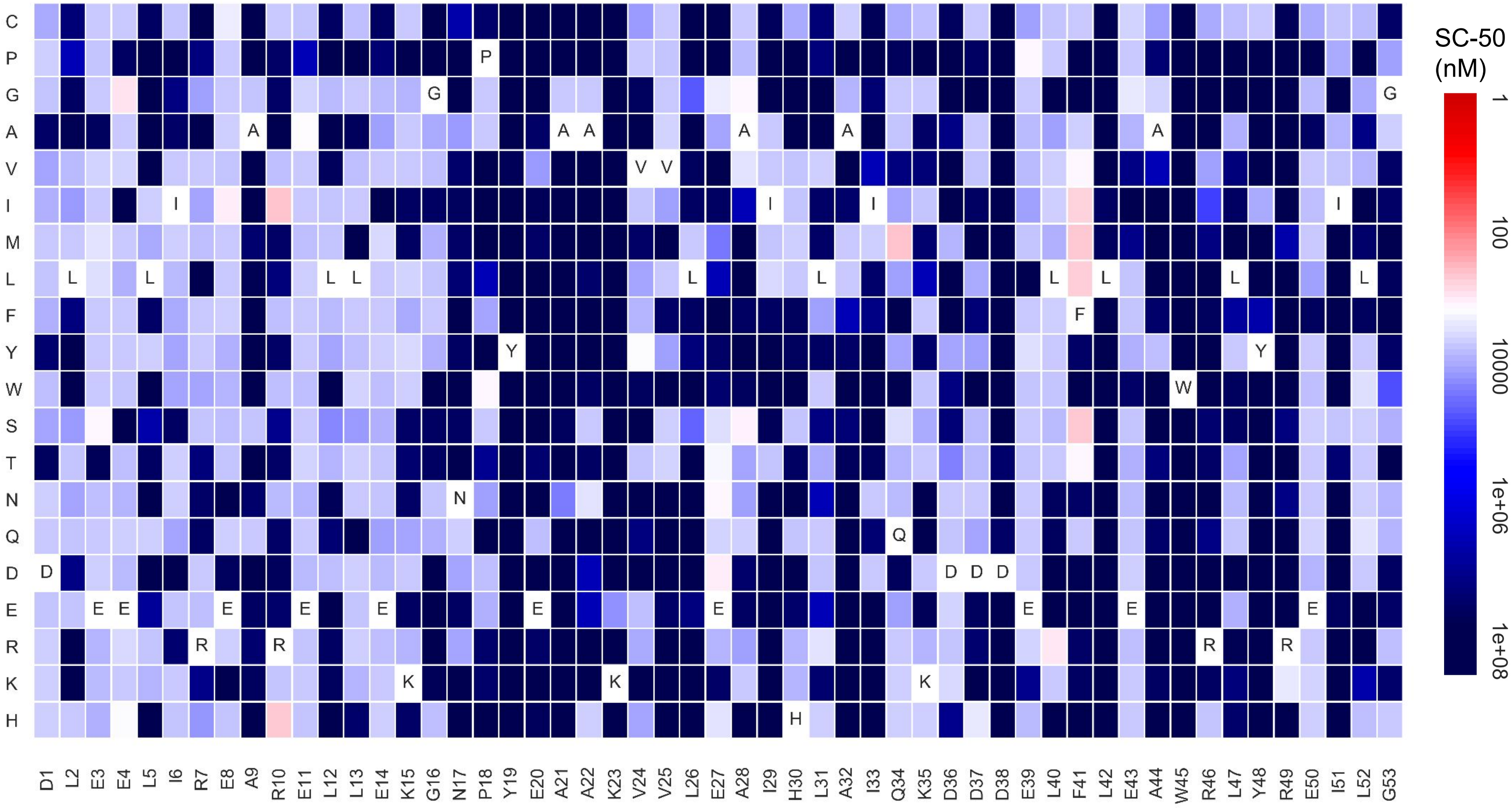

# Minibinder 6

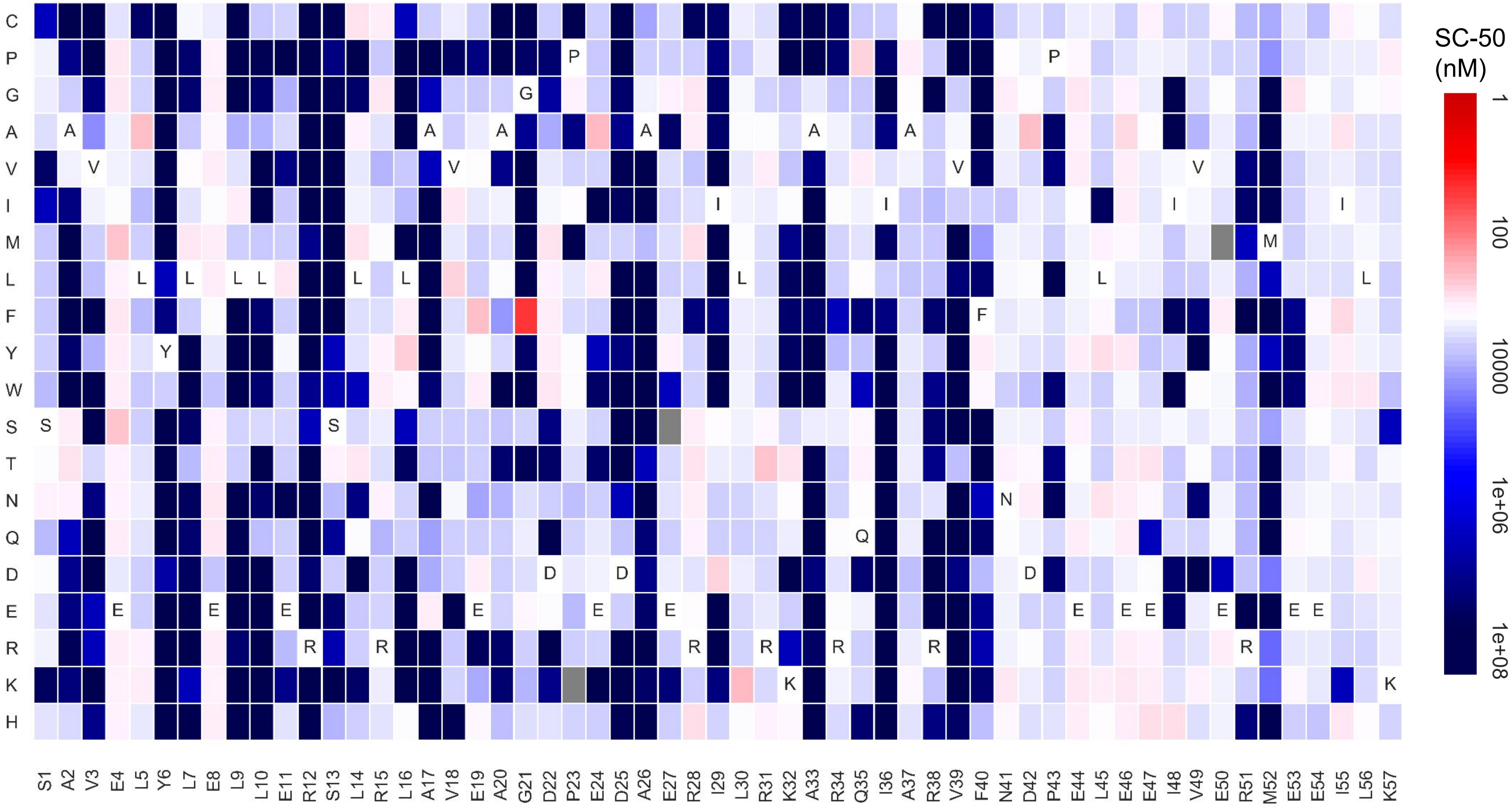

# Minibinder 7

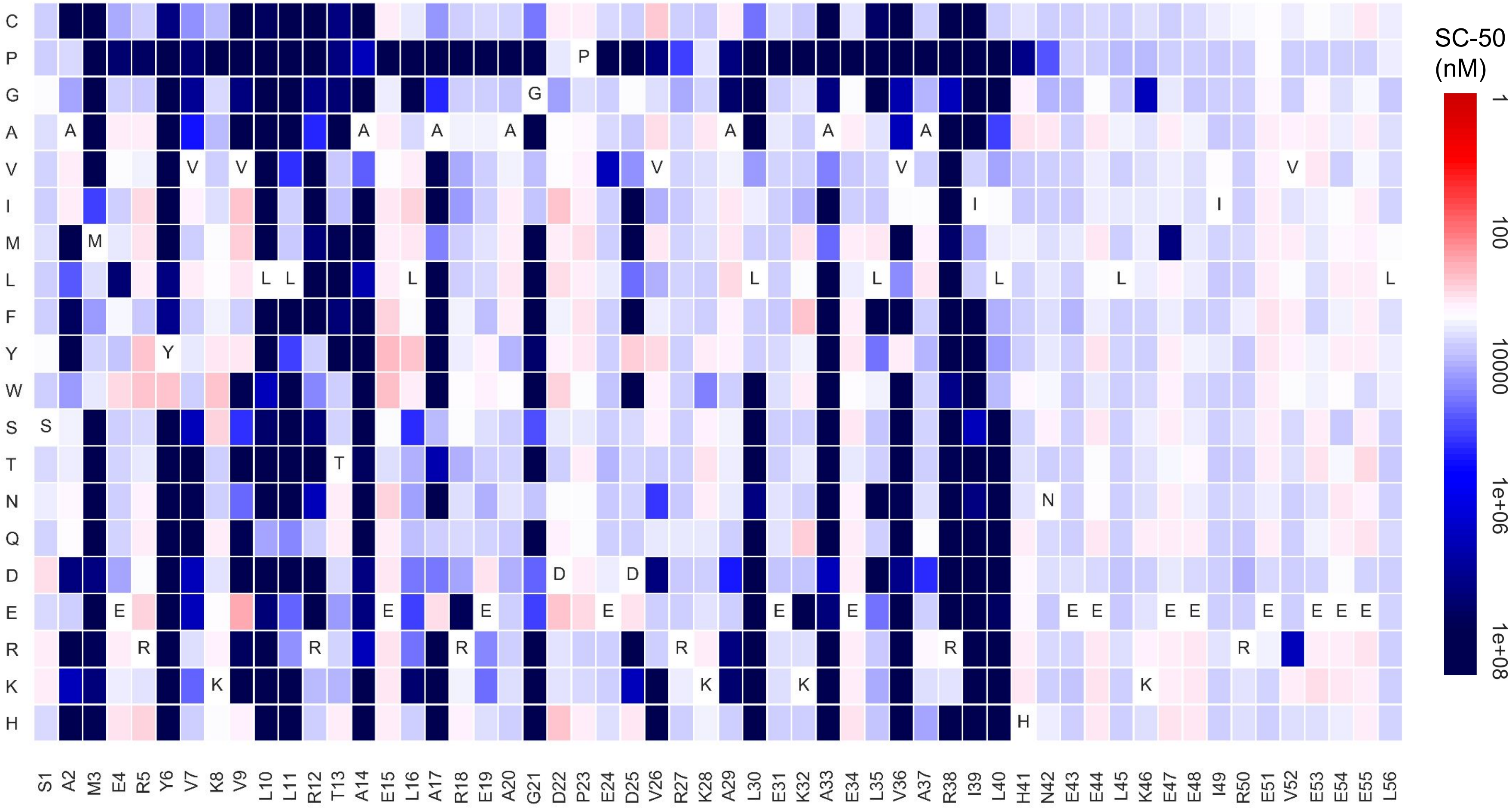

# Minibinder 8

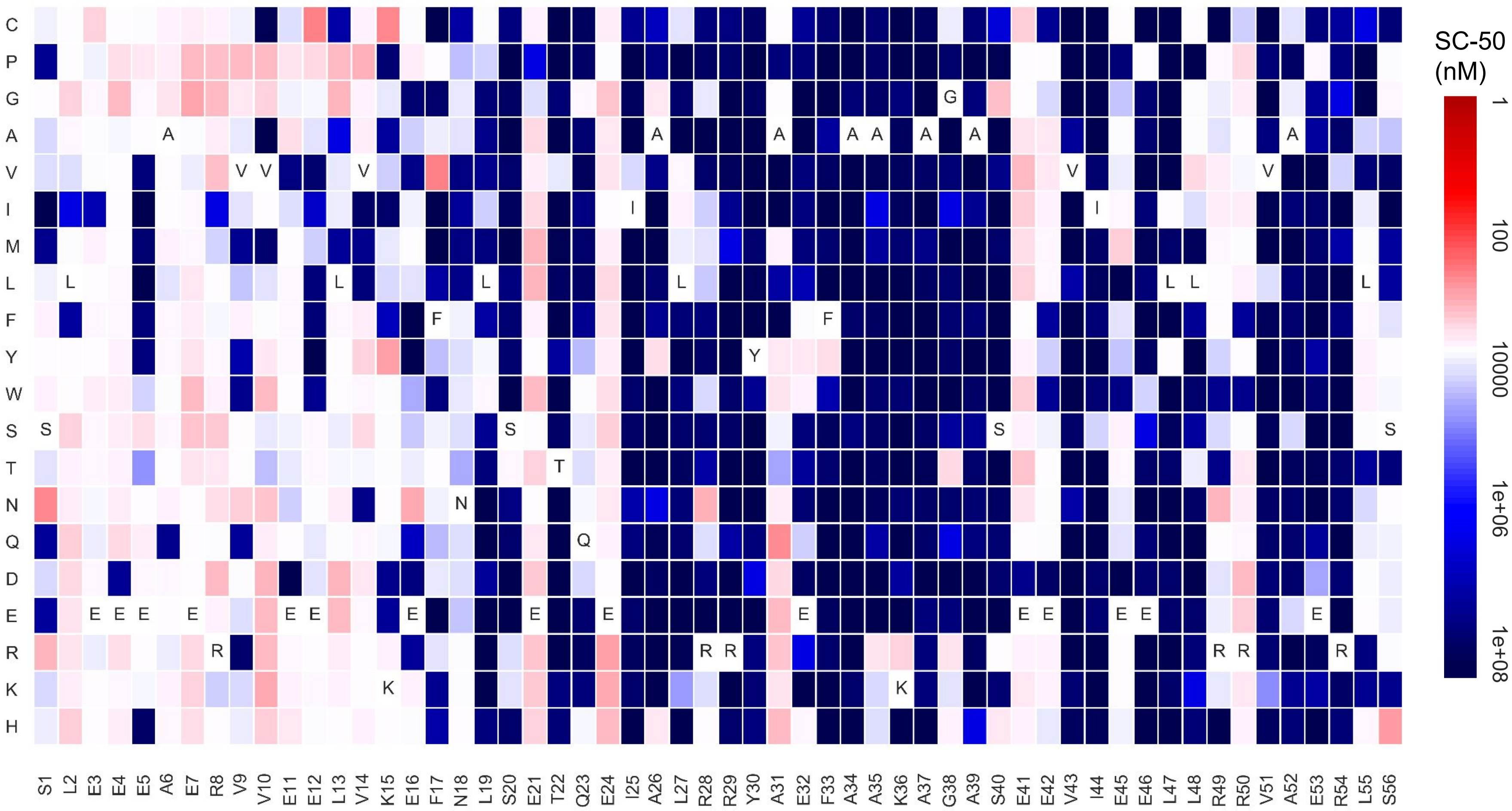

# Minibinder 9

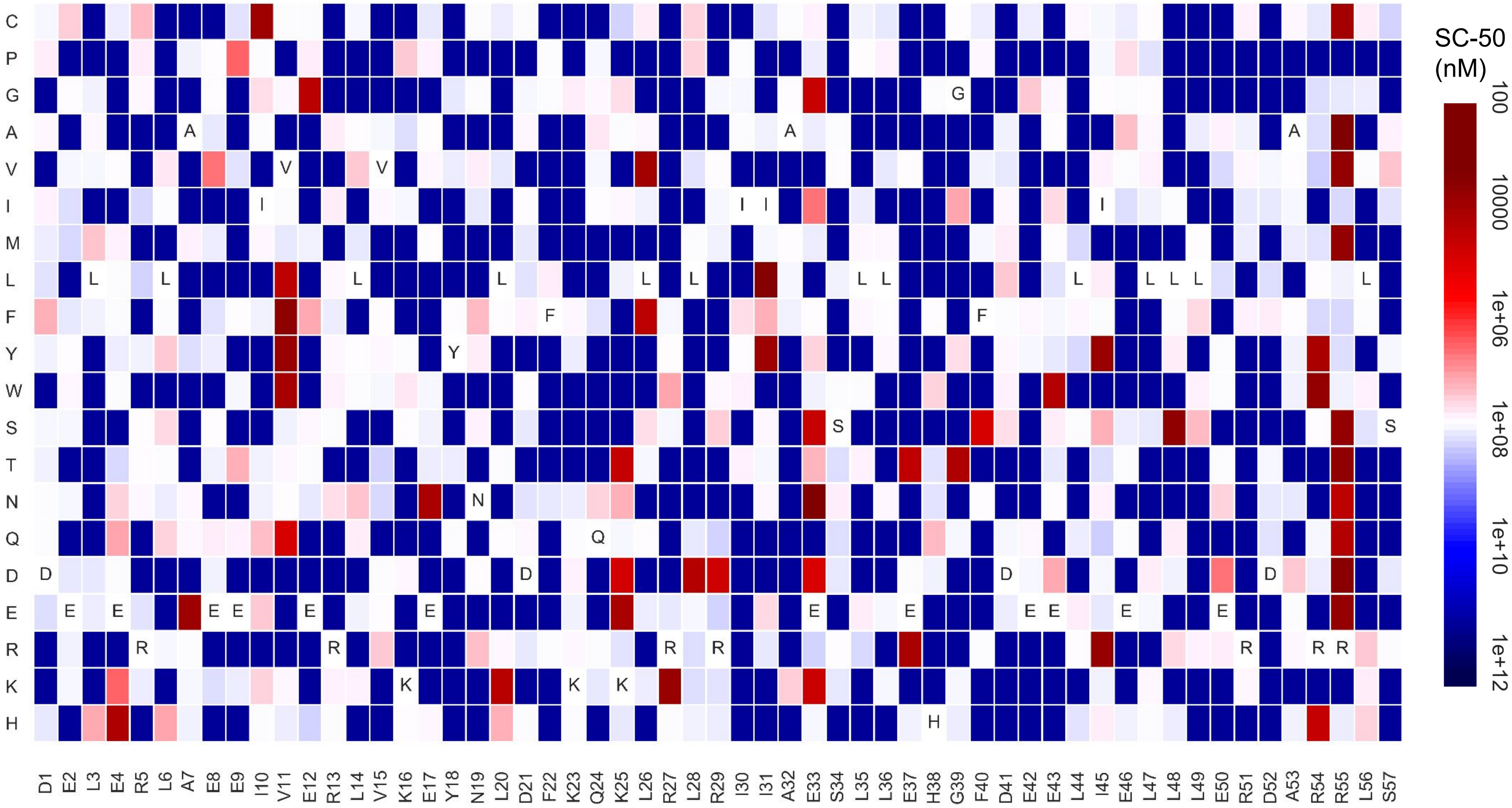

# Minibinder 10

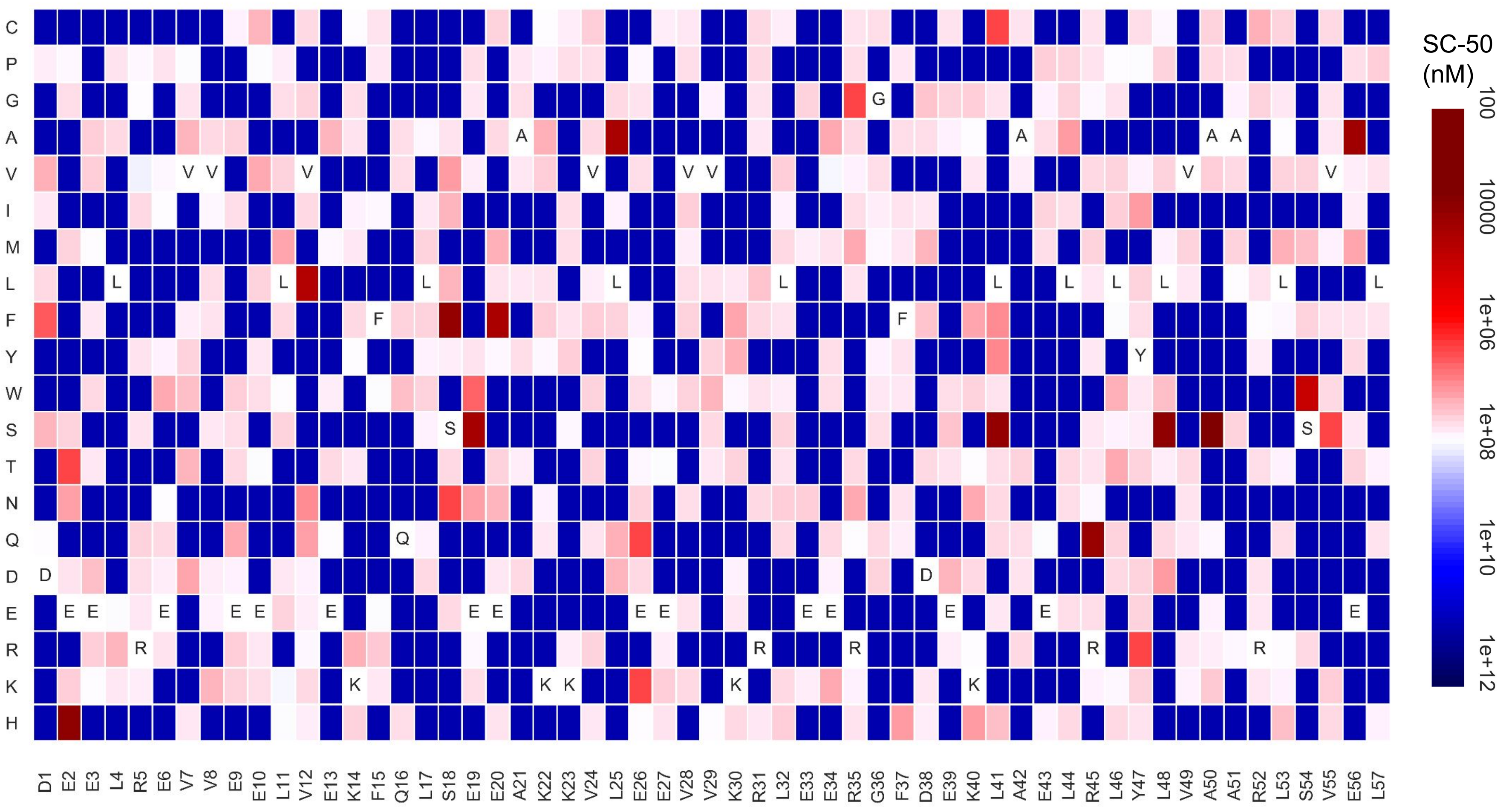

# Minibinder 11

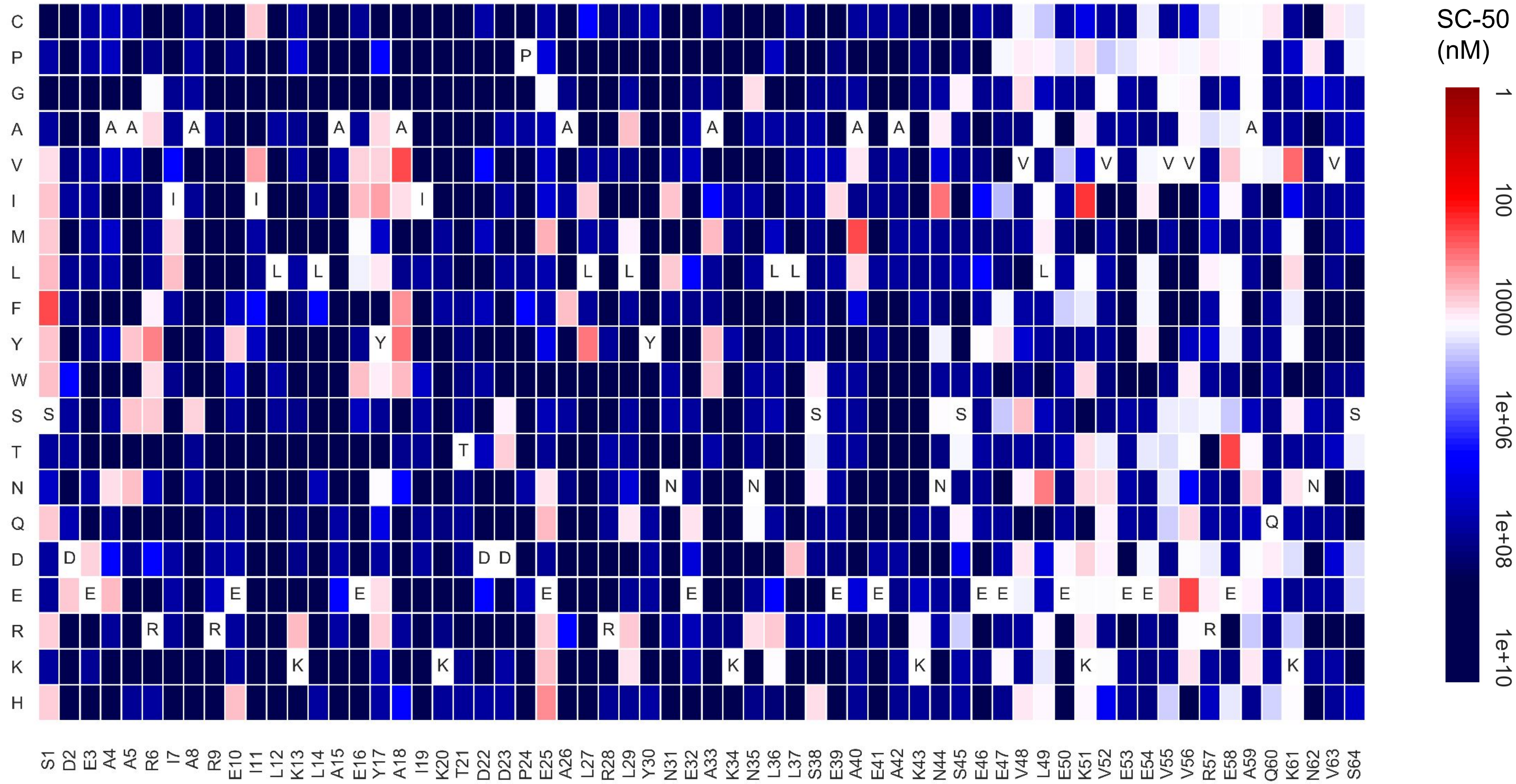

# Full gel from Extended Data Fig. 1

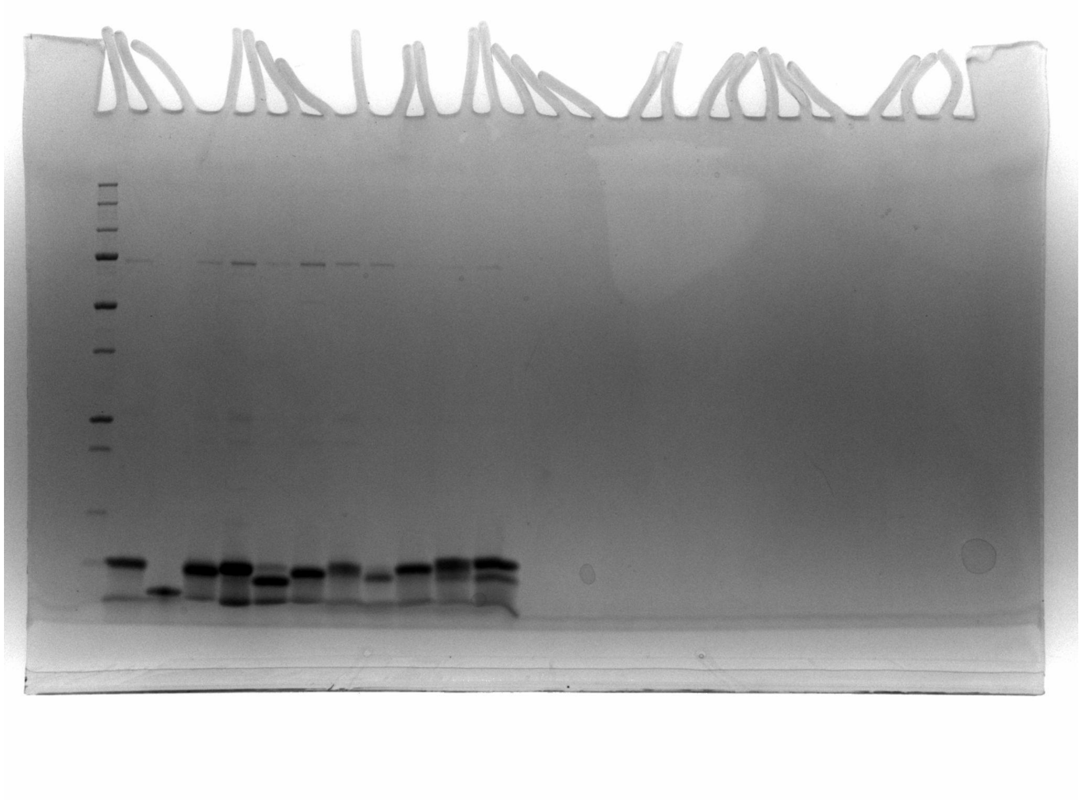

# Full gel from Extended Data Fig. 3

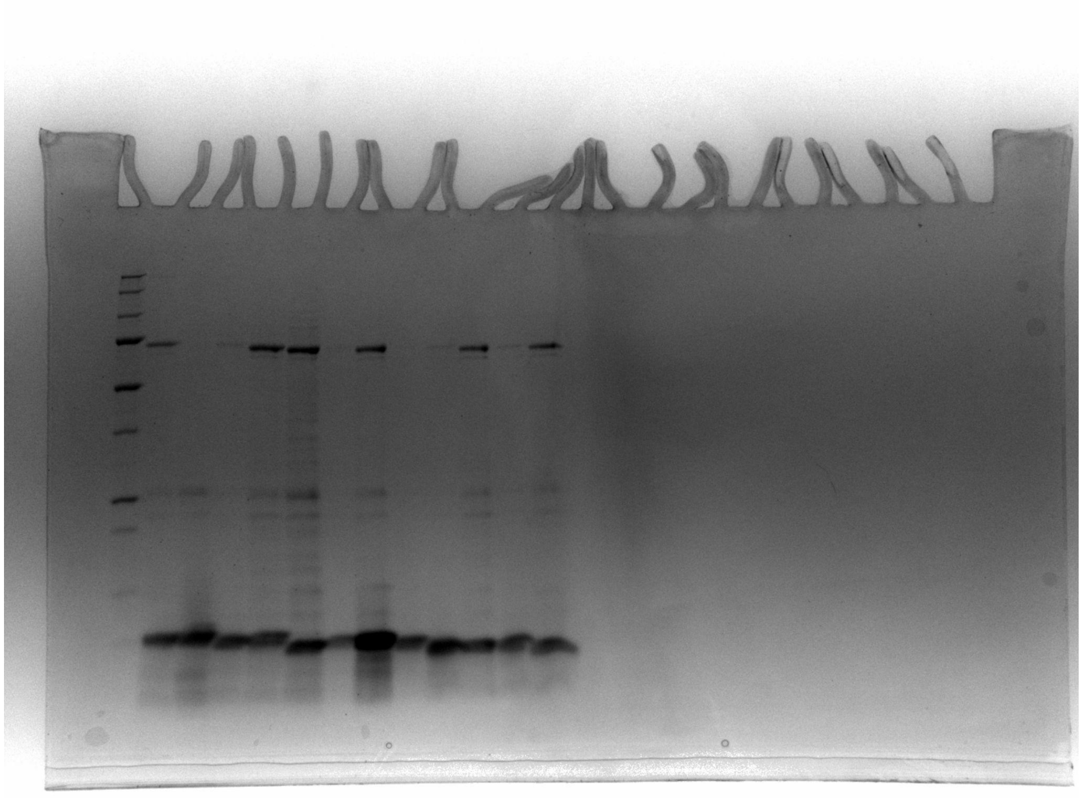

# Full gel from Extended Data Fig. 4

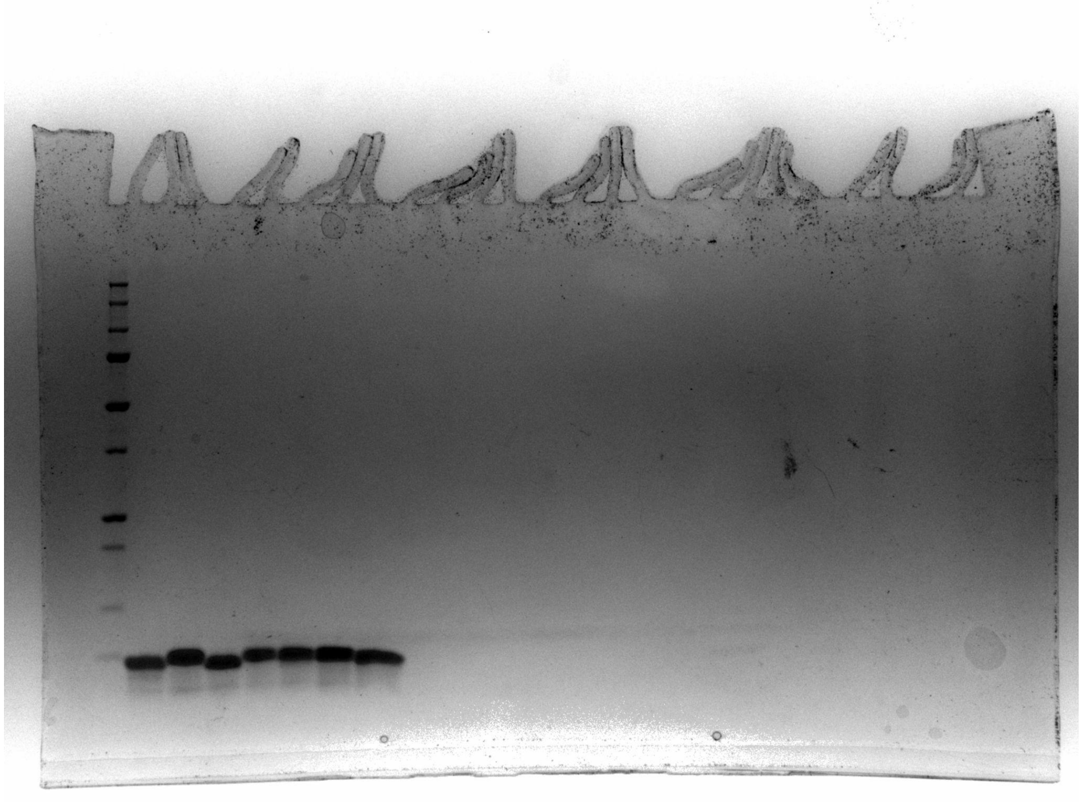

Supplement: Supplement 1 [file media-1.pdf]
