# Supplementary material for: De novo design of protein minibinder agonists of TLR3
Source: bioRxiv. 2024 Apr 18:2024.04.17.589973. Preprint. [Version 1] doi: 10.1101/2024.04.17.589973 (PMC11042314; doi:10.1101/2024.04.17.589973)
Supplement: 2 [file NIHPP2024.04.17.589973v1-supplement-2.pdf]

bioRxiv preprint doi: https://doi.org/10.1101/2024.04.17.589973; this version posted April 18, 2024. The copyright holder for this preprint (which was not certified by peer review) is the author/funder, who has granted bioRxiv a license to display the preprint in perpetuity. It is made available under aCC-BY 4.0 International license.

Supplementary Table 1. Amino acid sequences.

Construct*	Amino Acid Sequence
1	DR[**V**/I][**L**/Y]RA[**A**/R]AELAF[**K**/R]NLRID[**V**/I/L/M]EENDPEEVR[**Q**/S/T/Y]NLR[**H**/R]LET[**L**/Y]ARVLNDPEIERLVEEVKELLG
2	DEVWRILAQ[**M**/I][**T**/N][**H**/S]I[**L**/K/N/S/Q][**S**/N]HIDDPEE[**A**/N/S]YEV[**S**/D/N]RLF[**L**/Y]RVYELNDPEYARRALERAEEL
3	DN[**F**/Y]RYY[**V**/A/G]E[**Q**/N/S/Y]LLR[**S**/H/N/T]AEL[**L**/E/S]LEEGDPE[**E**/Y]AE[**L**/N/Q/R/S][**A**/R/S]L[**R**/H][**S**/N/Q]AKTVAR[**L**/I/V]ENDEELRRLVEELERRL
4	DD[**I**/N/T]FE[**F**/W]YIKYLIE[**A**/Q/N/T]AK[**R**/Y]AYEE[**G**/I]DDEAAENDLR[**T**/H]ARSAARVLGDEELRRLIEELERKI
5	DLEELIREARELLEKGNPYEAAK[**V**/Y]VLE[**A**/S]IHLAI[**Q**/M]K[**D**/R]DDE[**L**/R][**F**/I/L/M/S]LEAWRLYREILG
6	S[**A**/N/S/T]VELYLELLERS[**L**/M/T]R[**L**/F/Y]A[**V**/L]EAGDPEDAER[**I**/D][**L**/K]R[**K**/H/T]ARQIARVFNDPELEEIVERMEEILK
7	S[**A**/I/V]ME[**R**/H/Y]YVK[**V**/E]LLRTAE[**L**/Y]AREAG[**D**/E/H]PE[**D**/E/H/Y]VR[**K**/N/S/T][**A**/L]LE[**K**/F/Q]AE[L/M]VARILHNEELKEEIREVEEEL
8	SLEEEAERVVEELVKEFNLSET[**Q**/H]EI[**A**/H/Y]LRRY[**A**/E/H/R/Q]E[**F**/Y]AA[**K**/R]AGASEEVIEEL[**L**/V]RRVAERLS
9	DELERLAEEIVERLVKEYNLDFKQKLRLRIIAESLLEHGFDEELIELLLERDARRLS
10	DEELREVVERLVEKFQLSEEAKKVLEEVVKRLEERGFDEKLAKLRLYLVAARLSVEL
11	S[**D**/E]EA[**A**/Y]RIARE[**I**/V]LKLAEYAIKTDDPEALRLYNEAK[**N**/R]LL[**S**/H/N/R]EAEAKNSEEVLKKVEEVVRKAQKNVS
7.1	SAMEYYVKVLLRTAEYAREAGDPEDVRKALEKAELVARILHNEELKEEIREVEEEL
7.2	SAMEYYVKVLLRTAEYAREAGDPEYVRKALEKAELVARILHNEELKEEIREVEEEL
7.3	SAMEYYVKVLLRTAEYAREAGDPEYVRKALEQAELVARILHNEELKEEIREVEEEL
7.4	SAMEYYVNVLLRTAEYAREAGDPEDVRKALEKAELVARILHNEELKEEIREVEEEL
7.5	SAMEYYVKVLLRTAEYAREAGDPEDVRKALEQAELVARILHNEELKEEIREVEEEL
7.6	SAMEYYVKELLRTAEYAREAGDPEDVRKALEKAELVARILHNEELKEEIREVEEEL
7.7	SAMEYYVKELLRTAEYAREAGDPEYVRKALEKAELVARILHNEELKEEIREVEEEL
7.8	SAMEYYVKELLRTAEYAREAGDPEYVRKALEQAELVARILHNEELKEEIREVEEEL
7.9	SAMEYYVKELLRTAEYAREAGDPEYVRNALEQAELVARILHNEELKEEIREVEEEL

bioRxiv preprint doi: https://doi.org/10.1101/2024.04.17.589973; this version posted April 18, 2024. The copyright holder for this preprint (which was not certified by peer review) is the author/funder, who has granted bioRxiv a license to display the preprint in perpetuity. It is made available under aCC-BY 4.0 International license.

7.10	SAMEYYVKELLRTAEYAREAGEPEYVRNALEQAELVARILHNEELKEEIREVEEEL
7.11	SAMEYYVKELLRTAEYAREAGDPEDVRKALEQAELVARILHNEELKEEIREVEEEL
7.12	SAMEYYVKELLRTAEYAREAGDPEDVRNALEQAELVARILHNEELKEEIREVEEEL
7 KO	SAMERAVKVLLRTAELAREAGDPERVRKALEEAELVARILHNEELKEEIREVEEEL
8.1	SLEEEAERVVEELVKEFNLSRTQEIALRRYAEFAAKAGASEEVIEELLRRVAERLS
8.2	SLEEEAERVVEELVKEFNLSETQEIALRRYAEYAAKAGASEEVIEELLRRVAERLS
8.3	SLEEEAERVVEELVKEFNLSRTQEIALRRYAEYAAKAGASEEVIEELLRRVAERLS
8.4	SLEEEAERVVEELVKEFNLSRTQEIALRRYAEYAARAGASEEVIEELLRRVAERLS
8.5	SLEEEAERVVEELVKEFNLSRTQEIALRRYAEYAARATASEEVIEELLRRVAERLS
8.6	SLEEEAERVVEELVKEFNLSRTQEIALRRYAEYAARATASEEVIEELLRDVAERLS
8 KO	SLEEEAERVVEELVKEFNLSETQEIALRAAAEAAAKAGASEEVIEELLRRVAERLS
tetramer	**[BINDER]**GGSGGSGGSGGSGGSG[**BINDER]**GGSGGSGGSGGSGGSG[**BINDER]**GGSGGSGGSGGSGGSG[**BINDER]**
dimer	GSHENKQVEEILRLEKEIEDLQRMKERQELSLTEASLQKLQLEDKVEELLSKNYHLENEVARLKKLVGEGGSGGSGGSGGSGGSGGSGGSGGS[**BINDER]**
TLR3 ectodomain	MLLVNQSHQGFNKEHTSKMVSAIVLYVLLAAAAHSAFAKCTVSHEVADCSHLKLTQVPDDLPTNITVLNLTHNQLRRLPAANFTRYSQLTSLDVGFNTISKLEPELCQKLPMLKVLNLQHNELSQLSDKTFAFCTNLTELHLMSNSIQKIKNNPFVKQKNLITLDLSHNGLSSTKLGTQVQLENLQELLLSNNKIQALKSEELDIFANSSLKKLELSSNQIKEFSPGCFHAIGRLFGLFLNNVQLGPSLTEKLCLELANTSIRNLSLSNSQLSTTSNTTFLGLKWTNLTMLDLSYNNLNVVGNDSFAWLPQLEYFFLEYNNIQHLFSHSLHGLFNVRYLNLKRSFTKQSISLASLPKIDDFSFQWLKCLEHLNMEDNDIPGIKSNMFTGLINLKYLSLSNSFTSLRTLTNETFVSLAHSPLHILNLTKNKISKIESDAFSWLGHLEVLDLGLNEIGQELTGQEWRGLENIFEIYLSYNKYLQLTRNSFALVPSLQRLMLRRVALKNVDSSPSPFQPLRNLTILDLSNNNIANINDDMLEGLEKLEILDLQHNNLARLWKHANPGGPIYFLKGLSHLHILNLESNGFDEIPVEVFKDLFELKIIDLGLNNLNTLPASVFNNQVSLKSLNLQKNLITSVEKKVFGPAFRNLTELDMRFNPFDCTCESIAWFVNWINETHTNIPELSSHYLCNTPPHYHGFPVRLFDTSSCKDSAGGSHHHHHHGGSGLNDIFEAQKIEWHE

* For minibinders 1-11, the amino acids encoded by degenerate codons in the combinatorial libraries are listed in brackets, with the original amino acid bolded.

bioRxiv preprint doi: https://doi.org/10.1101/2024.04.17.589973; this version posted April 18, 2024. The copyright holder for this preprint (which was not certified by peer review) is the author/funder, who has granted bioRxiv a license to display the preprint in perpetuity. It is made available under aCC-BY 4.0 International license.

Supplementary Table 2. Association and dissociation rates for TLR3/minibinder interactions, determined by BLI

Minibinder	K_{on} ($M^{-1}s^{-1}$)	K_{off} (s^{-1})
1	N.D.*	N.D.
2	N.D.	N.D.
3	1.1E+04	4.5E-03
4	6.9E+03	1.7E-03
5	N.D.	N.D.
6	1.1E+04	4.9E-04
7	4.8E+03	2.1E-03
8	2.5E+03	3.0E-03
9	4.1E+03	6.3E-03
10	N.D.	N.D.
11	N.D.	N.D.
7.1	7.9E+03	3.4E-04
7.2	9.3E+03	1.6E-04
7.3	8.0E+03	2.3E-04
7.4	1.0E+04	3.6E-04
7.5	1.6E+04	1.8E-05
7.6	5.0E+04	1.2E-03
7.7	1.9E+04	5.9E-04
7.8	2.0E+04	7.7E-04
7.9	2.9E+04	2.7E-03
7.10	3.0E+04	5.7E-03
7.11	3.0E+04	1.4E-03
7.12	4.0E+04	4.4E-03
8.1	2.8E+03	1.9E-03
8.2	2.1E+03	1.3E-03
8.3	2.9E+03	6.5E-04
8.4	2.9E+03	6.5E-04
8.5	3.3E+03	5.6E-04
8.6	4.5E+03	1.7E-04

*N.D. indicates no binding data.

bioRxiv preprint doi: https://doi.org/10.1101/2024.04.17.589973; this version posted April 18, 2024. The copyright holder for this preprint (which was not certified by peer review) is the author/funder, who has granted bioRxiv a license to display the preprint in perpetuity. It is made available under aCC-BY 4.0 International license.

Supplementary Table 3. Cryo-EM data collection, refinement, and validation statistics

	TLR3/minibinder 7.7	TLR3/minibinder 8.6
**Data collection and processing**		
Magnification	130,000	130,000
Voltage (kV)	300	300
Electron exposure ($e^-/Å^2$)	68.9	69.8
Defocus range (μm)	-0.8 – -2.2	-0.8 – -2.0
Pixel size (Å)	0.664	0.664
Symmetry imposed	C1	C1
Initial particle images (no.)	6,608,485	2,955,749
Final particle images (no.)	739,755	510,475
Map resolution (Å)	2.88	2.88
FSC threshold	0.143	0.143
**Refinement**		
Initial model used	AF2 prediction	AF2 prediction
Map resolution (Å)	2.88	2.88
FSC threshold	0.143	0.143
Map sharpening B factor ($Å^2$)	0	0
**Model composition**		
Non Hydrogen atoms	5,845	5,990
Protein residues	703	719
Ligands	14	15
**B factors ($Å^2$)**		
Protein	75.39	64.40
Ligand	77.43	70.91
**R.m.s. deviations**		
Bond lengths (Å)	0.26	0.26
Bond angles (°)	0.51	0.51
**Validation**		
MolProbity score	2.33	2.15
Clashscore	11	11
Poor rotamers (%)	0	0
**Ramachandran plot**		
Favored (%)	88	87
Allowed (%)	12	13
Outliers (%)	0	0
